# Task-shifting point-of-care CD4+ testing to lay health workers in HIV care and treatment services in Namibia

**DOI:** 10.4102/ajlm.v6i1.643

**Published:** 2017-09-18

**Authors:** Francina Kaindjee-Tjituka, Souleymane Sawadogo, Graham Mutandi, Andrew D. Maher, Natanael Salomo, Claudia Mbapaha, Marytha Neo, Anita Beukes, Justice Gweshe, Alexinah Muadinohamba, David W. Lowrance

**Affiliations:** 1 Directorate of Special Programs, Ministry of Health and Social Services, Windhoek, Namibia; 2 US Centers for Disease Control and Prevention, Windhoek, Namibia

## Abstract

**Introduction:**

Access to CD4+ testing remains a common barrier to early initiation of antiretroviral therapy among persons living with HIV/AIDS in low- and middle-income countries. The feasibility of task-shifting of point-of-care (POC) CD4+ testing to lay health workers in Namibia has not been evaluated.

**Methods:**

From July to August 2011, Pima CD4+ analysers were used to improve access to CD4+ testing at 10 selected public health facilities in Namibia. POC Pima CD4+ testing was performed by nurses or lay health workers. Venous blood samples were collected from 10% of patients and sent to centralised laboratories for CD4+ testing with standard methods. Outcomes for POC Pima CD4+ testing and patient receipt of results were compared between nurses and lay health workers and between the POC method and standard laboratory CD4+ testing methods.

**Results:**

Overall, 1429 patients received a Pima CD4+ test; 500 (35.0%) tests were performed by nurses and 929 (65.0%) were performed by lay health workers. When Pima CD4+ testing was performed by a nurse or a lay health worker, 93.2% and 95.2% of results were valid (*p* = 0.1); 95.6% and 98.1% of results were received by the patient (*p* = 0.007); 96.2% and 94.0% of results were received by the patient on the same day (*p* = 0.08). Overall, 97.2% of Pima CD4+ results were received by patients, compared to 55.4% of standard laboratory CD4+ results (*p* < 0.001).

**Conclusions:**

POC CD4+ testing was feasible and effective when task-shifted to lay health workers. Rollout of POC CD4+ testing via task-shifting can improve access to CD4+ testing and retention in care between HIV diagnosis and antiretroviral therapy initiation in low- and middle-income countries.

## Introduction

In 2012, United Nations Programme on HIV/AIDS (UNAIDS) reported that only about 8 million of the 15 million people in low- and middle-income countries (LMIC) in need of antiretroviral therapy (ART) were receiving ART by the end of 2011.^1^ In the LMIC of sub-Saharan Africa, many barriers exist to early initiation of ART among eligible HIV-positive patients. Of these barriers, limited access to CD4+ testing for determination of ART eligibility is one of the most common.^2,3,4,5,6,7^ A recent systematic review of 28 published studies examining the retention of patients in sub-Saharan Africa in HIV care between testing and treatment found that the median proportion of patients retained between HIV diagnosis and receipt of CD4+ test results was 59% (range: 35% – 88%).^8^

Implementation of rapid, point-of-care (POC) CD4+ testing in HIV care and treatment (HCT) settings has been shown to increase the number of patients who undergo and receive the results of CD4+ testing, and has the potential to improve retention between HIV diagnosis and ART initiation.^9^ The Pima® POC CD4+ analyser (Alere) has been validated in the field against standard-of-care, laboratory-based platforms^10,11,12,13^ and was listed as a World Health Organization (WHO) prequalified diagnostic in November 2011.^14^ Recent field-based studies in Mozambique and South Africa demonstrated that POC testing with the Pima CD4+ enabled clinics to rapidly stage patients on-site after enrolment into care, which reduced opportunities for pre-treatment loss to follow-up.^15,16^ POC CD4 testing has also been successfully deployed in mobile, community-based and household settings in South Africa.^17,18^

Task-shifting to meet human resources for health needs is recommended by WHO.^19^ Because POC CD4+ testing using the Pima CD4+ analyser does not require a laboratory and is relatively easy to perform, the task of performing CD4+ tests in the HCT setting could potentially be shifted from nurses and laboratory technicians to non-professional or lay health workers.

In Namibia, lay health workers in the ‘community counsellors’ cadre have been trained and certified by the Namibia Ministry of Health and Social Services (MOHSS) to provide health facility-based HIV testing and counselling. Roll-out and scale-up of POC CD4+ testing in HCT settings could follow a similar model. We assessed the feasibility of POC CD4+ testing performed by nurses or lay health workers and the effectiveness of POC CD4+ testing in reducing result turn-around time (TAT) and increasing the proportion of patients who receive their CD4+ test results.

## Methods

### Ethical considerations

Prior to implementation, the study protocol was approved by the Research Committee of the Namibia Institute of Pathology and the US Centers for Disease Control and Prevention. The analysis of the data collected for this study was approved as ‘non-research’ by the US Centers for Disease Control and Prevention and the Office of the Permanent Secretary of the MOHSS of Namibia. Informed patient consent is not required for receipt of routine services (including CD4 count testing) performed at MOHSS facilities and no personally identifiable information was available to the researchers; thus, written informed consent was not collected from patients.

### Study design

In July 2011, the Namibia MOHSS began a multi-phased rollout of POC CD4+ testing using the Pima CD4+ analyser in select public sector HCT clinics throughout the country. Prior to the first phase of the rollout of POC CD4+ testing, nurses and lay health workers received theoretical and practical training in use of the Pima analyser. Trainees were required to pass a written examination designed by the device manufacturer (Alere, Waltham, Massachusetts, United States) prior to performing testing in this study. From July 2011 to August 2011, Pima CD4+ POC tests were used for routine CD4+ testing for both baseline assessment of ART eligibility and routine ART monitoring among adult patients (age ≥ 15 years) receiving HCT services at 10 public clinics scattered across Namibia selected purposively by virtue of having an on-site laboratory. Lay health worker and nurse testers were also purposively selected for testing during this study by virtue of availability at these 10 clinics.

### Testing procedures

All patients eligible to receive standard-of-care CD4 testing at the participating clinics during the study period were consecutively sampled. Pima CD4+ testing was performed by trained nurses or lay health workers and both cadres were represented at all 10 sites. Patient management actions including initiation of ART, referral for pre-ART follow-up and requisition of further laboratory tests were initiated by clinicians based on the Pima CD4+ result of each patient. For the purpose of comparing some CD4+ results, TAT and patient receipt of results for Pima CD4+ and standard testing methods, venous blood was collected by nurses from approximately 10% of participants and sent to the laboratory for standard CD4+ testing on either the EPICS XL or FC500 (Beckman-Coulter, Brea, California, United States); results were returned to the clinic upon availability.

### Data collection

The following data were recorded by nurses or lay health workers in study-specific registers: location, date and time of specimen collection, testing cadre, validity (i.e. ‘valid’ or ‘invalid’) of each Pima CD4+ test result, time of Pima CD4+ test result availability, time of test result disclosure to the patient, Pima CD4+ result and patient demographic information, including age, sex and reason for CD4+ testing (i.e. ART eligibility assessment or ART monitoring). A questionnaire was sent to all nurses and lay health workers by the study staff to rate their experience with using the Pima CD4+ test towards the end of the study period. The rating categories ranged from very easy, easy, fair, difficult to very difficult. The times of specimen collection and patient receipt of results for standard laboratory CD4+ testing were also collected. Patient management actions initiated by clinicians following receipt of POC CD4+ results were recorded.

### Analysis

Basic patient demographic and clinical characteristics were calculated and stratified by sex and reason for CD4+ testing. The feasibility of task-shifting POC CD4+ testing to lay health workers was assessed by calculating the following for both nurses and lay health workers: percentage of valid Pima CD4+ test results produced, percentage of Pima CD4+ test results received by patients overall and received by patients on the same day as testing, median Pima CD4+ result TAT and percentage of eligible patients (i.e. Pima CD4+ result ≤ 350 cells/µL) who initiated ART at the same or next clinic visit.

All percentages were calculated at the 95% confidence level. Two-proportion z-tests and Pearson’s chi-square or the Mann-Whitney test (for median result TAT only), were used to test for significant differences between the tester cadres. The effectiveness of POC CD4+ testing was assessed by calculating the percentage of patients who received their Pima CD4+ or standard laboratory CD4+ results and the median and interquartile range (IQR) for the TAT for both CD4+ testing methods. Two-proportion z-tests and Pearson’s chi-square or the Wilcoxon matched-pairs test (for median TAT results), were used to test for significant differences between the Pima CD4+ testing and standard laboratory testing methods. The Wilcoxon test, which is non-parametric, was used due to the asymmetrical distribution of CD4+ TAT results.

## Results

Overall, 1429 patients (68% women) received a Pima CD4+ test during the study period (Table 1). Of these, 407 patients (28.5%) received Pima CD4+ tests for an ART eligibility assessment and 1022 patients (71.5%) received Pima CD4+ tests for ART monitoring. Pima results ranged from 11–641 cells/µL overall. The median Pima CD4+ result among patients tested for an ART eligibility assessment was 233 cells/µL (IQR: 128–365) among women and 202 cells/µL (IQR: 82–363) among men. Based on their Pima CD4+ results, 69.5% of patients were eligible to initiate ART (i.e. ≤ 350 cells). The median Pima CD4+ result among patients tested for ART monitoring was 338 cells/µL (IQR: 221–462) among women and 218 cells/µL (IQR: 142–358) among men.

**TABLE 1:** Demographic and clinical characteristics of patients who received point-of-care Pima CD4+ tests performed by nurses or lay health workers, Namibia, July–August 2011.

Variable	Reason for CD4+ testing	
	ART eligibility assessment	ART monitoring
Sex: *n* (%)		
Women	253 (26.0)	719 (74.0)
Men	154 (34.0)	303 (64.0)
Age: Median years (IQR)		
All	34 (28–40)	36 (29–42)
Women	31 (26–38)	34 (28–41)
Men	37 (32–41)	38 (32–45)
Pima CD4+ result: Median cells/µL (IQR), range		
All	222 (111–363), 18–641	306 (182–439), 11–627
Women	233 (128–365)	338 (221–462)
Men	202 (82–363)	218 (142–358)
Eligible to initiate ART by Pima result, i.e. CD4+ ≤ 350 cells/µL: *n* (%)		
All	283 (69.5)	
Women	107 (37.8)	
Men	176 (62.2)	

ART, antiretroviral therapy; IQR, interquartile range.

Of the 1429 Pima CD4+ tests performed, 500 (35.0%) were performed by nurses and 929 (65.0%) by lay health workers (Table 2). Sixty-one per cent (61%) of testers were lay health workers. Results for nurses and lay health workers were similar for the validity of Pima CD4+ test results and for receipt of results by patients on the same day as testing. A statistically significant difference between cadres was found for overall receipt of Pima CD4+ results by the patient (95.6% for nurses, 98.1% for lay health workers; *p* = 0.007). The median result TAT was 20 min when performed by nurses and 21 min when performed by lay health workers (*p* = 0.39). Among the 283 patients who were eligible to initiate ART based on the results of their Pima CD4+ test, data on patient management action taken either at the same visit when Pima CD4+ testing was performed or at the next follow-up visit were available for 226 (79.9%). Among these, 7.7% who were tested by nurses and 5.6% who were tested by lay health workers (*p* = 0.5) were documented as initiating ART at the same eligibility assessment visit or at the next follow-up visit. The majority of both nurses and lay health workers rated the Pima CD4+ test favourably for ease of use.

**TABLE 2:** Comparison of outcomes for point-of-care Pima CD4+ testing performed by nurses or by lay health workers, Namibia, July–August 2011.

Variable	Testing cadre		*p*^†^
	Nurses	Lay health workers	
Pima CD4+ performed: *n* (%)	500 (35.0)	929 (65.0)	---
Valid Pima CD4+ performed: % (CI)^‡^	93.2 (91.0–95.0)	95.2 (94.0–96.6)	0.1
Pima CD4+ results received by patient: % (CI)	95.6 (93.8–97.4)	98.1 (97.2–99.0)	0.007
Pima CD4+ results received by patient same day: % (CI)^§^	96.2 (94.5–97.9)	94.0 (92.5–95.6)	0.08
Pima CD4+ results turnaround time: median minutes (IQR)	20 (18–27)	21 (18–26)	0.39
Eligible patient (i.e. Pima CD4 ≤ 350) initiated ART^¶^	7.7 (1.1–14.2)	5.6 (2.0–9.2)	0.5
Tester rated Pima CD4+ favourably: % (CI)	96.6 (94.0–97.5)	94.7 (92.9–95.9)	0.4

ART, antiretroviral therapy; IQR, interquartile range.

† Significance tested by two-proportion z-test and Pearson’s chi-square or Mann-Whitney test (for median result TAT only).

‡ All confidence intervals (CI) are at the 95% level.

§ Percentage calculated only among patients who received Pima result (*n* = 1389).

¶ Percentage calculated only among patients eligible to initiate ART who received results and had data on subsequent patient management action available (*n* = 226).

Among patients who received a Pima CD4+ test, 1389 (97.2%) patients received their test results, of whom 1317 (92.2%) received their results on the same day (Table 3). Of the 128 specimens collected for CD4+ testing by standard laboratory methods, 71 (55.4%) of results had been received by patients by the end of the study period. The median TAT for Pima CD4+ test results was 20 min (IQR: 18–27), whereas the median TAT for standard laboratory CD4+ test results was 4 days (IQR: 2–8).

**TABLE 3:** Comparison of outcomes for point-of-care Pima CD4+ testing with outcomes for standard laboratory CD4+ testing method, Namibia, July–August 2011.

Variable	Method of CD4+ test		*p*^†^
	Pima	Standard laboratory	
Patient received CD4+ result: % (CI)^‡^^,^^§^	97.2 (96.3–98.1)	55.4 (46.7–64.1)	< 0.001
Patient received CD4+ result same day: %(CI)	92.2 (90.8–93.6)	4.7 (0.9–8.3)	< 0.001
Results turnaround time: median (IQR)	20 min (18–27)	4 days (2–8)	

† Significance tested by two-proportion z-test and Pearson’s chi-square test.

‡ All confidence intervals (CI) are at the 95% level.

§ Pima, *n* = 1429; Standard laboratory, *n* = 128.

## Discussion

This study demonstrated that POC CD4+ testing was feasible and effective when performed by lay health workers. Whether performed by nurses or lay health workers, POC CD4+ testing reduced TATs for laboratory results by a median of 4 days compared to TATs for standard laboratory methods and greatly increased receipt of CD4+ test results by patients. With more than 95% of patients receiving same-day CD4+ results, the potential for attrition between HIV diagnosis and ART initiation was greatly reduced. When compared to the 59% median for the percentage of patients who were retained between HIV diagnosis and receipt of CD4+ test results in Rosen and Fox’s systematic review,^8^ our results indicate that retention between HIV diagnosis and receipt of CD4+ results could be greatly improved by task-shifting POC CD4+ testing to nurses and lay health workers. Almost all nurses and lay health workers in our pilot rated the POC Pima CD4+ favourably on ease-of-use questionnaires; thus, this pilot provides additional evidence supporting the feasibility of task-shifting POC CD4+ testing to nurses and lay health workers in this environment.

Extensive evidence supports such task-shifting in HCT settings in LMIC in sub-Saharan Africa.^20,21,22,23,24,25,26,27^ In several countries in sub-Saharan Africa that are affected by a generalised HIV epidemic and a critical shortage of skilled health care providers, scale-up of voluntary HIV testing and counselling has been facilitated by the utilisation of non-professional or lay health worker cadres.^28,29^ Results from the current study show that additional efficiencies can be gained by task-shifting POC CD4+ testing to lay health workers. Although current WHO recommendations for expanding access to POC CD4+ testing only recommend that POC diagnostics (with the exception of HIV rapid tests) be performed by first-tier clinical staff, our results support the idea that task-shifting of POC CD4+ testing and other diagnostics to lay health workers is appropriate.

In our study, POC CD4+ testing greatly increased the percentage of patients who received their CD4+ test results. However, among patients eligible to initiate ART based on their Pima CD4+ results, fewer than 10% actually initiated ART at the baseline study visit or the next follow-up visit. Among these, 7.7% who received Pima CD4+ results from tests performed by nurses and 5.6% (*p* = 0.5) who received results from lay health workers initiated ART at the same eligibility assessment visit or at the next follow-up visit. The possible reasons for the slightly higher percentage who initiated ART soon after a PIMA CD4+ test done by a nurse rather than a lay health worker are not clear. However, this finding may indicate that nurses who conduct the test and review the results are more likely to initiate the patient on ART themselves, thereby eliminating the potential barrier of referral to a doctor for ART initiation. In contrast, lay health workers are not qualified to interpret CD4 results, nor can they initiate patients on ART. They can only refer patients for review of results and clinical management, steps which create a potential bottleneck for ART initiation.

At the time this study was conducted in 2011, the Namibia guidelines^30^ recommended routine baseline tests for everyone prior to initiation of ART. This has since slightly changed with the 2014 national guidelines^31^ which adopted the WHO 2013 guideline recommendation^32^ of same-day ART initiation for some eligible patients. While still recommending baseline tests for all patients, the Namibia guidelines now recommend that all HIV-positive pregnant and breastfeeding women should be initiated on ART on the same day that ART eligibility is established, even in the absence of baseline results. This indicates that, apart from pregnant and breastfeeding women for whom baseline tests are now not mandatory to initiate ART, POC CD4+ testing alone may not remove all barriers to early initiation of ART and opportunities for pre-treatment loss to follow-up may persist without additional steps. Similarly, low rates of early ART initiation following receipt of Pima CD4+ results were observed by Jani et al.^15^ in their studies of Pima CD4+ in Mozambique. One contributing factor may be that additional diagnostics for the purpose of determining appropriate first-line regimens, including serum creatinine testing for contraindication to tenofovir-containing regimens^33,34^ and alanine transaminase or hepatitis B virus serology for contraindication to nevirapine-containing regimens, are recommended, and may be performed over several visits before ART is initiated. In Namibia, and in most LMIC, these tests are typically performed through standard laboratory-based methods in centrally located laboratories away from the POC so that, like standard laboratory CD4+ testing, patients must return at a later date to receive their results. POC diagnostics for hepatitis B virus,^35^ alanine transaminase,^36,37^ serum creatinine^38^ and other tests are available and should be evaluated alongside POC CD4+ testing to further reduce opportunities for pre-treatment loss resulting from sub-optimal access to the diagnostics used to determine ART eligibility and appropriate first-line regimens.

### Limitations

Several factors may limit the external validity of results from this study. Selection bias may have been introduced by purposive selection of clinics that have an on-site laboratory. Clinics with a robust infrastructure may have had better trained or more skilled staff than clinics with weaker infrastructure. Selection bias may also have been introduced by purposive selection of testers. Lay health workers and nurses were selected by virtue of their availability at the selected clinics during the study period. The lay health worker to nurse ratio in public clinics across Namibia may not reflect the lay health worker to nurse ratio in our study.

Finally, we did not assess the accuracy of Pima CD4+ results in relation to the standard laboratory-based platforms, nor did we compare accuracy of testing between lay health workers and nurses. Although the accuracy of CD4+ testing is paramount for determining eligibility and effective monitoring, the investigators felt that the accuracy of the Pima analyser had been sufficiently validated in previously published studies. Therefore, assessing the accuracy of Pima CD4+ testing was not included among the objectives or measures of this study.

### Conclusion

In this study, we demonstrated that POC CD4+ testing was feasible and effective when performed by lay health workers and nurses within an HCT service delivery setting, leading to high levels of patient receipt of results and greatly improved TATs for CD4+ results. By removing one of the major sources of pre-treatment loss to follow-up, POC CD4+ testing has great potential to increase early ART initiation. However, additional diagnostic barriers, particularly the ancillary tests required to determine the appropriate first-line regimen after determination of ART eligibility, need to be removed in order to further increase linkage to treatment. Task-shifted POC CD4+ testing should be more widely implemented as an important tool to both increase access to treatment and improve the efficiency of ART services.
